# Precision net ultrafiltration dosing in continuous kidney replacement therapy: a practical approach

**DOI:** 10.1186/s40635-023-00566-8

**Published:** 2023-11-28

**Authors:** Raghavan Murugan, Kianoush Kashani, Paul M. Palevsky

**Affiliations:** 1 The Program for Critical Care Nephrology, Department of Critical Care Medicine, University of Pittsburgh School of Medicine, Pittsburgh, Pennsylvania United States of America; 2 The Center for Research, Investigation, and Systems Modeling of Acute Illness (CRISMA), Department of Critical Care Medicine, University of Pittsburgh School of Medicine, Pittsburgh, Pennsylvania United States of America; 3 Division of Nephrology and Hypertension, Division of Pulmonary and Critical Care Medicine, Department of Medicine, Mayo Clinic, Rochester, Minnesota United States of America; 4 Renal and Electrolyte Division, Department of Medicine, University of Pittsburgh School of Medicine, Pittsburgh, Pennsylvania United States of America; 5 Kidney Medicine Section, Veterans Affairs Pittsburgh Healthcare System, Pittsburgh, Pennsylvania United States of America

**Keywords:** Net ultrafiltration, Acute kidney injury, Continuous kidney replacement therapy, Net fluid removal, Ultrafiltration rate

## Introduction

Fluid overload occurs in more than two-thirds of critically ill patients with acute kidney injury (AKI) receiving kidney replacement therapy (KRT) and is independently associated with morbidity and mortality [1, 2]. International consensus guidelines recommend extracorporeal net fluid removal when a life-threatening fluid overload occurs in a patient with oliguric AKI refractory to diuretics [3–5]. However, the optimal method of net fluid removal during KRT remains to be determined, and there is global variation in clinical practice [6–10]. Some clinicians propose using net fluid balance as a target for the fluid removal [11, 12], while others suggest using the prescribed vs. delivered net fluid removal gap [13, 14]. However, all these methods influence the net fluid removal rate (*i.e.,* net ultrafiltration rate [UF_NET_] rate) one way or another during KRT.

Several observational studies show that the UF_NET_ rate when adjusted for the patient actual body weight (ABW) has a “J” shaped association with mortality in critically ill patients with AKI receiving continuous kidney replacement therapy (CKRT) [15–20]. Patients who received UF_NET_ rates of 1.01 to 1.75 mL/kg/h had the lowest mortality and KRT dependence compared to patients who received slower (< 1.01 mL/kg/h) or faster (> 1.75 mL/kg/h) rates [15–18]. In addition, UF_NET_ rates > 1.75 mL/kg/h were associated with an increased risk of cardiac arrhythmias requiring treatment [15]. As randomized trials have not been conducted, the causality between UF_NET_ rate and mortality is unclear.

### Definition of net ultrafiltration

During CKRT, the CKRT machine continuously removes plasma water from the patient's intravascular compartment. This process is known as ultrafiltration (UF) [21, 22]. The ultrafiltration rate (*i.e.,* UF rate) is the rate at which plasma water is removed from blood per unit of time (mL/h) [23]. The term UF rate connotes only the volume removed from the patient's intravascular compartment. It excludes the removal of any obligatory fluids (*i.e.,* dialysate and replacement fluids) administered during CKRT [22, 23]. The UF_NET_ rate represents the net fluid removed from the intravascular compartment over and beyond any intravenous fluids directly infused into the patient simultaneously outside the CKRT machine. For instance, in an 80-kg patient with a UF rate of 160 mL/h who receives 80 mL/h of continuous intravenous infusion, the delivered UF_NET_ rate is 80 mL/h (*i.e.,* 160 minus 80) or 1.0 mL/kg/h.

### Importance of weight-based net ultrafiltration dosing

First, since UF_NET_ is a form of controlled hypovolemia, it represents a form of cardiovascular stress [24, 25]. During UF_NET_, fluid removal from the intravascular compartment is accompanied by vascular refill because of fluid shifts from the extravascular into the intravascular compartment [26]. Vascular refilling depends on not only the UF_NET_ rate, but also the degree of fluid overload, transcapillary hydrostatic and osmotic pressure gradients, dialysate sodium concentration, administration of colloidal or hypertonic solutions, endothelial glycocalyx and basement membrane, extracellular matrix, lymphatic flow, and systemic inflammation [22, 26–28]. When UF_NET_ is performed at a higher rate than the vascular refill rate, the total circulating blood volume declines, resulting in intravascular hypovolemia, decreased preload, cardiac output, and hypotension [22, 29].

Thus, intravascular volume status must be frequently assessed during UF_NET_ using point-of-care ultrasound (POCUS) such as venous excess ultrasound score (VExUS) or pulse pressure/ stroke volume variation (PPV/SVV), especially in obese individuals in whom volume status assessment can be challenging [30, 31]. Moreover, UF_NET_ must only be performed during the stabilization and de-escalation phases of shock when the patient is hemodynamically stable and the goal is to achieve negative fluid balance. However, UF_NET_ may occasionally be helpful during salvage and optimization phases when the patient has refractory pulmonary edema [5, 32].

Second, observational studies indicate an association between UF_NET_ rate and mortality only when the UF_NET_ rate is adjusted for patient body weight (*i.e.,* mL/kg/h rather than mL/h). For instance, UF_NET_ of 100 mL/h in a patient weighing 100 kg and receiving no continuous infusions is only 1.0 mL/kg/h. Meanwhile, in a patient who weighs only 50 kg, the UF_NET_ rate is 2.0 mL/kg/h, suggesting differing cardiovascular stress depending upon the patient's weight for any given UF_NET_ rate. Thus, UF_NET_ must be dosed like a drug or effluent dose based on the patient's body weight [33, 34]. Herein, we describe a practical method for precise UF_NET_ dosing during CKRT.

### A practical approach to precision net ultrafiltration dosing

Before commencing UF_NET_, we suggest discontinuing all unnecessary IV fluids and double concentrating medications to minimize infused volume. We also suggest confirming excess intravascular volume status using POCUS or other methods of volume assessment. The hourly UF_NET_ dosing during CKRT is based on three essential steps: (i.) determining patient weight; (ii.) selecting a desired UF_NET_ dosing rate range (e.g., 1.0–2.0 mL/kg/h); and (iii.) calculating the hourly continuous infusions and fluid balance in any given hour.

### Step 1: determine the patient body weight

We propose using predicted body weight (PBW) to set the UF_NET_ rate during CKRT. PBW is estimated using a nomogram based on the patient’s height and sex [35]. We selected PBW to standardize the dosing of UF_NET_ for any given patient independent of variations in ABW and quantify the cardiovascular stress in terms of UF_NET_ dose. We also suggest using PBW for the following reasons: (i.) PBW is free of confounding by daily variations in patient ABW due to fluid balance [36], catabolism from critical illness [37], and other measurement errors [36]; (ii.) PBW has been shown to approximate ideal medication dosing weight in males and females [35]; and (iii.) PBW could be precisely determined in the patient before initiating fluid removal.

The PBW may be calculated using the following equations:

$${\text{PBW }}\left( {{\text{kilograms}}} \right){\text{ }} = {\text{ 45}}.{\text{5 }} + {\text{ 2}}.{\text{3 }}\left[ {{\text{height }}\left( {{\text{inches}}} \right){\text{ }}{-}{\text{ 6}}0} \right]$$

for female patients, and,

$${\text{PBW }}\left( {{\text{kilograms}}} \right){\text{ }} = {\text{ 5}}0{\text{ }} + {\text{ 2}}.{\text{3 }}\left[ {{\text{height }}\left( {{\text{inches}}} \right){\text{ }}{-}{\text{ 6}}0} \right]$$

for male patients [38].

We recommend not using the patient ABW because precise premorbid ABW may not be known in critically ill patients. Moreover, ABW documented in medical records during previous or current hospitalization may not be reliable because of confounding by the underlying illness that led to hospitalization (*e.g.,* volume depletion from sepsis may result in underestimation, and fluid overload from heart failure may result in overestimation). Furthermore, using ABW will require daily changes in UF_NET_ dosing as weight decreases secondary to fluid removal.

Among obese patients, we still recommend using PBW because the adipose tissue of obese individuals exhibits a substantial reduction in blood vessel density, disrupted blood flow, and endothelial dysfunction [39–43]. Thus, the cardiovascular stress during UF_NET_ is less likely to vary as adiposity increases. Since obesity might be associated with more significant fluid overload proportional to the fatty tissue, obese patients with severe fluid overload may require a prolonged duration of fluid removal for any constant UF_NET_ rate based on PBW.

### Step 2: determine the desired UF_NET_ rate dosing range

We recommend selecting a UF_NET_ rate dosing range for the patient. While the optimal UF_NET_ rate is unknown, we recommend cautiously using higher UF_NET_ rates until more research is available. Higher UF_NET_ rates may be used if the risk of not rapidly treating fluid overload (e.g., severe respiratory distress due to cardiogenic pulmonary edema) outweighs the risk of complications from higher UF_NET_ rates [15]. In an ongoing clinical trial (NCT05306964), study ICUs are randomized to restrictive or liberal approaches to UF_NET_ [44]. In the restrictive arm, fluid removal is between 0.5 and 1.5 mL/kg/h of PBW, and in the liberal arm, between 2.0 and 5.0 mL/kg/h of PBW. In both arms, fluid removal starts at 0.5 mL/kg/h and gradually increases to maintain between the assigned target UF_NET_ rate ranges, as tolerated by the patient hemodynamics. The UF_NET_ rates corresponding to these dosing ranges have been used widely in clinical practice [9].

### Step 3: calculate hourly continuous fluid infusion and fluid balance

Since the UF_NET_ rate represents the removal of net intravascular volume, continuous intravenous patient infusions must be accounted for in the calculation. For example, in a patient with a PBW of 80 kg, a delivered UF_NET_ rate of 1.5 mL/kg/h would be 120 mL/h (80 × 1.5) if the patient receives no intravenous fluids. However, if the patient receives 80 mL/h of intravenous infusion in the current hour, the patient-delivered UF_NET_ rate is only 40 mL/h (i.e., 120—80 = 40 mL/h) or 0.5 mL/kg/h. The fluids infused may be that of intravenous fluids, medications, blood, plasma, and combinations thereof. Enteral and oral feedings and gastrointestinal and drain losses can also be included in determining the precise UF_NET_ rate. However, how much the gastrointestinal fluid shifts directly impact circulating intravascular volume in the same hour and thus influence the delivered UF_NET_ dose is complex and depends on several factors such as the rate of fluid absorption and loss from the gastrointestinal tract, the patient volume status, and rate of capillary refill. Since we developed this protocol primarily to determine the precise UF_NET_ rate for iatrogenic fluid infusions, clinician discretion is recommended on which fluids to include (*e.g.,* chest tube and abdominal drains) in the calculations when there are complex fluid shifts. Box 1 shows a case example of a hypothetical patient with UF_NET_ dosing based on the above method.

**Table:** 

Case study of precision net ultrafiltration rate calculation and dosing during CKRT	
A 60-year-old female patient is admitted to the emergency room in septic shock secondary to ischemic small bowel. She is hypotensive and required 5 L of fluid resuscitation in the emergency room and initiation of norepinephrine. She subsequently underwent an exploratory laparotomy, small bowel resection, and abdominal washout and her bowels are left in discontinuity. She received another 3 L of fluid bolus during the surgery to maintain hemodynamics. At ICU admission, her fluid balance was positive for 8 L. 24 h following ICU admission, she developed oliguric acute kidney injury with urine output of 100 mL in the last 24 h. Her urine analysis shows muddy brown casts. Her serum creatinine increased from a baseline of 0.8 mg/dL to 2.0 mg/dL. She was therefore started on CKRT for oliguric acute kidney injury and fluid management.	
Her body weight at hospital admission is 80 kg, and her height is 63 inches. She receives a continuous infusion of 60 mL/h of intravenous TPN, 4 mL/h of propofol (40 mg/hour), 2 mL/h of fentanyl (100 mcg/h), and 18 mL/h of norepinephrine (0.12 mcg/kg/min).	
**Precision UF**_**NET**_ **rate calculation and delivery during CKRT:**	
**Step 1**: Based on the gender-specific nomogram, her predicted body weight (PBW) is 52.4 kg	
**Step 2**: Desired UF_NET_ rate = 1.0—2.0 mL/kg/h	
**Step 3**: Continuous intravenous patient infusion = 84 mL/h (60 + 4 + 2 + 18)	
Based on the above information, her UF_NET_ rate range of 1.0 to 2.0 mL/kg/h would be between 136.4 mL/h (52.4 + 84) and 188.8 (104.8 + 84) mL/h.	
CKRT UF_NET_ can be started at a rate of 0.5 mL/kg/h (*i.e.,* 136.4/2 = 68.2 mL/h) and gradually increased to 188.8 mL/h as tolerated by hemodynamics. The net fluid removal rate is then continued and varied between 136.4 and 188.8 mL/h as tolerated by the patient. If the patient infusion of 84 mL/h changes at any time, then the new UF_NET_ rate must be recalculated based on the above method to deliver the UF_NET_ rate precisely. If the patient has stoma output or other fluid losses, they can be incorporated into the calculation. A worksheet ( Additional file 1) can be used to calculate the precise net fluid removal rate	

### Use of clinical decision support system

A clinical decision support system (CDSS) can automate the calculation of the UF_NET_ rate and may facilitate easy implementation. For example, if one enters the patient's height and sex to determine the PBW, preselects the desired UF_NET_ rate range, and enters the continuous infusions and fluid balance per hour, a CDSS can calculate the UF_NET_ rate. The recommended UF_NET_ rate can then be set on the CKRT machine. The CDSS algorithm may be incorporated into a computer (*e.g.,* iPad, laptop, or desktop) application (“app”), electronic medical records, and eventually into the CKRT machine software. Herein, we have developed a UF_NET_ rate calculator worksheet (Additional file 1) that helps clinicians to precisely dose and track the delivered UF_NET_ rate during CKRT since this information may only be routinely available in some electronic health records.

## Conclusions

In summary, precision delivery of UF_NET_ dosing during CKRT can be achieved based on patient body weight, intended rate of net fluid removal, and continuous infusion of intravenous fluids, and hourly fluid balance.

### Supplementary Information

**Additional file 1.** Worksheet for determining and tracking the precise UF_NET_ rate during continuous kidney replacement therapy.

## Data Availability

Not applicable.
